# Fragmentation of maternal, child and HIV services: A missed opportunity to provide comprehensive care

**DOI:** 10.4102/phcfm.v8i1.1240

**Published:** 2016-12-02

**Authors:** J. Lyn Haskins, Sifiso P. Phakathi, Merridy Grant, Ntokozo Mntambo, Aurene Wilford, Christiane M. Horwood

**Affiliations:** 1 Centre for Rural Health, University of KwaZulu-Natal, South Africa; 2 School of Applied Science, University of KwaZulu-Natal, South Africa

## Abstract

**Background:**

In South Africa, coverage of services for mothers and babies in the first year of life is suboptimal despite high immunisation coverage over the same time period. Integration of services could improve accessibility of services, uptake of interventions and retention in care.

**Aim:**

This study describes provision of services for mothers and babies aged under 1 year.

**Setting:**

Primary healthcare clinics in one rural district in KwaZulu-Natal, South Africa.

**Methods:**

All healthcare workers on duty and mothers exiting the clinic after attending well-child services were interviewed. Clinics were mapped to show the route through the clinic taken by mother–baby pairs receiving well-child services, where these services were provided and by whom.

**Results:**

Twelve clinics were visited; 116 health workers and 211 mothers were interviewed. Most clinics did not provide comprehensive services for mothers and children. Challenges of structural layout and deployment of equipment led to fragmented services provided by several different health workers in different rooms. Well-child services were frequently provided in public areas of the clinic or with other mothers present. In some clinics mothers and babies did not routinely see a professional nurse. In all clinics HIV-positive mothers followed a different route. Enrolled nurses led the provision of well-child services but did not have skills and training to provide comprehensive care.

**Conclusions:**

Fragmentation of clinic services created barriers in accessing a comprehensive package of care resulting in missed opportunities to provide services. Greater integration of services alongside immunisation services is needed.

## Introduction

Providing high-quality maternal, child and women’s health (MCWH) care is vital to improving health outcomes. However, in South Africa many mothers and babies lack access to quality services during the postnatal period and beyond. Malnutrition remains an important cause of morbidity and mortality in young children, and coverage of key interventions, particularly for HIV care, is suboptimal for both mothers and babies in the months after birth. Adoption of prevention of mother-to-child transmission (PMTCT) guidelines, which recommend that all HIV-positive pregnant women start lifelong antiretroviral therapy (ART) at the time of HIV diagnosis, increases the importance of retention in care and provision of a comprehensive package of services for mothers and children at primary health care (PHC) level.

In the antenatal period, PMTCT services have been successfully integrated into routine care in well-established antenatal clinics^1^ leading to almost universal coverage of HIV testing among pregnant women and high uptake of ART among women identified as HIV-positive.^2^ However, it has been difficult to ensure that HIV-positive mothers are retained in care and continue taking ART, and that their babies receive early infant diagnosis and initiate ART^3^ where appropriate in the postnatal period.^4^ As ongoing HIV care devolves to PHC facilities, clinics must be prepared to adopt innovative strategies to deliver a high standard of care and to retain mothers and babies in care.^5^

Integration of health services has been widely advocated as a key strategy to improve access to care, efficiency and cost-effectiveness of service provision, and patient satisfaction.^6,7^ Service integration can be defined as the bringing together of services and activities that share common goals^8^ and has received widespread support from policy makers.^9,10,11,12^ The policy framework in South Africa supports provision of integrated PHC services giving equal attention to promotive, preventive, curative and rehabilitative services.^13^ The South African Department of Health’s recent ‘Re-engineering Primary Health Care‘ strategy has increased interest in the concept of integrated delivery systems.^14^

The aim of an integrated MCWH service at PHC level would be to provide a comprehensive package of essential preventive and curative services, linked to immunisation services, for mothers and children at a single visit. A comprehensive package of MCWH care in the well-child setting should include: for the baby, post natal examinations, growth monitoring, immunisation, provision of Vitamin A supplementation, deworming medication, and developmental screening; for the mother, family planning, dual protection with condom use, tuberculosis, sexually transmitted disease and cervical screening; for both mother–baby pair, HIV care for both positive and negative mothers. This could improve coverage of services, and reduce mortality and morbidity. Immunisation coverage among infants in South Africa is high,^15^ so this setting provides an ideal opportunity to provide integrated MCWH services. Linking additional interventions to a robust immunisation programme with high coverage rates has been shown to lead to rapid increases in the coverage of the linked intervention.^16^

In this study, we investigated the provision of MCWH services at PHC well-child clinics in one district in KwaZulu-Natal (KZN), and we describe a detailed picture of service delivery at PHC level.

## Research methods and design

### Study design

This study was a cross-sectional descriptive survey conducted in 12 PHC clinics. The study was conducted in preparation for an intervention phase to improve integration of MCWH health services currently underway in these clinics.

### Study setting

The study was conducted in one predominantly rural district in KZN, South Africa. It comprises five sub-districts with three district hospitals and one regional hospital. The district has a population of approximately 700 000.

### Study population and sampling strategy

Twelve clinics were purposively selected from 24 clinics in the district based on immunisation numbers: 12 clinics with the highest number of first immunisations, based on district health information system (DHIS), data were selected. Data collection was conducted by a team of experienced researchers over the course of one day for each of 12 clinics. All health workers on duty and all mothers attending for well-child services were requested to participate.

### Data collection

Data were collected using a variety of different data collection tools and techniques (Table 1). Operational managers (OMs) of clinics were interviewed about the range of services the clinic provided for mothers and children. A floor plan of the clinic was drawn to record the clinic layout and deployment of health workers in clinical areas on that day. The flow of mother–baby pairs through the clinic was observed and plotted on the floor plan. Each consulting room in the clinic was then observed, and equipment and services provided for mothers and children in that consulting room were recorded using a structured checklist.

**TABLE 1:** Sources of data and data collection methods.

Data collection activity	Source of data	Data collected	Type of data
Clinic mapping	Operational manager interview Floor plan drawing	Staff establishment Opening hours MCWH and HIV services provided Cadre of health worker providing the service Flow of mother and infants during their visit to the clinic	Reported Observed
Review of activities in clinic	Health worker working in each consulting room	Equipment available Services provided	Reported and observed
Exit interviews with mothers	Mothers attending for immunisation of infants	Services provided during the clinic visit	Reported
Health worker interview	All health workers on duty on the day of data collection	MCWH and HIV services provided in the previous month Relevant training received	Reported

MCHW, maternal, child and women’s health.

All health workers on duty on the day of data collection were interviewed (including professional nurses, enrolled nurses, enrolled nursing assistants and lay health workers) using a structured questionnaire to determine their role in provision of MCWH services, relevant training they had received.

All mothers attending the clinic for well-child services on the day of data collection were interviewed upon leaving the clinic, to determine coverage of services.

### Data analysis

Data were entered in EpiData 3.1, and analysis of descriptive data was conducted using Stata 13 for Windows.

### Ethical considerations

Ethical approval was obtained from the Biomedical Ethics Review Committee at the University of KwaZulu-Natal (BEO26/12). Permission to undertake the study was obtained from the KZN Department of Health. All participants provided written informed consent.

## Results

Twelve clinics were visited between September and December 2012. OMs in all clinics reported providing a wide range of maternal and child health services. However, not all clinics provided a comprehensive range of services: some clinics reported that they did not provide certain services, such as postnatal checks on mothers 6 weeks postpartum, and hearing assessments on babies. Only in 1 of the 12 clinics did the OMs report providing the full range of services (Table 2).

**TABLE 2:** Services provided in the clinics as well as the equipment needed to provide the service.

Maternal/child health service *N* = 12	No. of clinics reporting this service was provided	Supplies/equipment required to provide this service	Supplies/equipment available in immunisation room	Supplies/equipment available in the clinic
Postnatal check mothers at 6 days/6 weeks	8	Bed/couch	6	12
Postnatal check babies at 6 days/6 weeks	12	Baby-weighing scale	6	12
Immunisation	12	Cooler box containing immunisations	12	12
Vitamin A supplementation	12	Vitamin A capsules	7	12
Deworming	12	Supply of deworming medicine	6	11
Infant feeding counselling	12	Counselling flip charts	0	7
Sick-child consultation	12	IMCI chart booklet	3	12
Weighing of babies	12	Baby scale	5	12
Measuring the length of babies	7	Length board or tape measure	1	6
Measuring head circumference of babies	8	Tape measure	2	12
Developmental screening	11	n/a	n/a	n/a
Hearing checks for babies	5	n/a	n/a	n/a
Providing family planning	12	Oral contraceptives Injectable contraceptives	3 9	12 9
Cervical screening	12	Vaginal speculum + slides + functioning light	4	11
PCR test for babies	12	PCR testing kit	4	12
TB screening	12	n/a	n/a	n/a
STI screening	12	n/a	n/a	n/a
Providing condoms	12	Male condoms	5	12

PCR, Polymerase chain reaction; TB, tuberculosis; STI, sexually transmitted infections.

Equipment to provide all the services was available in most clinics (Table 2). However, equipment was frequently scattered around the clinic and not available in the rooms where health workers were providing that particular service. None of the clinics had equipment available to provide the full range of services in the immunisation room. Medications, including oral contraceptives and antiretroviral drugs, were kept with the professional nurse in all clinics.

### Organisation of mother–baby services

Upon arrival at the clinic, mothers and babies seeking well-child services received directions from a staff member. Although these directions determined the pathway by which mother–baby pairs proceeded through the clinic and, therefore, which services they could access, directions were given in an ad hoc way, usually by a junior member of the clinic staff, and without clear guidance. Staff members who undertook this role on the day of data collection varied and included enrolled nurses (five clinics), enrolled nursing assistants (one clinic), community health workers (one clinic), the receptionist (four clinics) and the security guard (one clinic).

In 11 of 12 clinics, babies were undressed and weighed in public areas with other patients present. This was done in the waiting room in seven clinics, in the immunisation room in three clinics and in the vital signs room (where routine observations like temperature and blood pressure are done for all patients) in two clinics. Weighing of babies was done by enrolled nurses in eight clinics, enrolled nursing assistant in two clinics, a community health worker in one clinic and either an enrolled nurse or the receptionist in one clinic.

In all 12 participating clinics there was a designated room where immunisations were always given (immunisation room), and enrolled nurses gave the immunisations. In seven clinics, immunisations were routinely given in a room with other mothers present. In five clinics, mother–baby pairs were seen privately, either in a separate room (four clinics) or behind a curtain (one clinic).

In seven clinics, mother–baby pairs were routinely seen by a professional (registered) nurse during their immunisation visit, in the other five clinics immunisation services were provided without reference to a professional nurse.

In all 12 clinics, the flow of the mother–baby pairs was different for HIV-positive mothers compared to uninfected mothers. In 9 of 12 clinics HIV-positive mothers attending for well-child services routinely saw a professional nurse, and in three clinics these mothers routinely saw a HIV counsellor.

**FIGURE 1:**
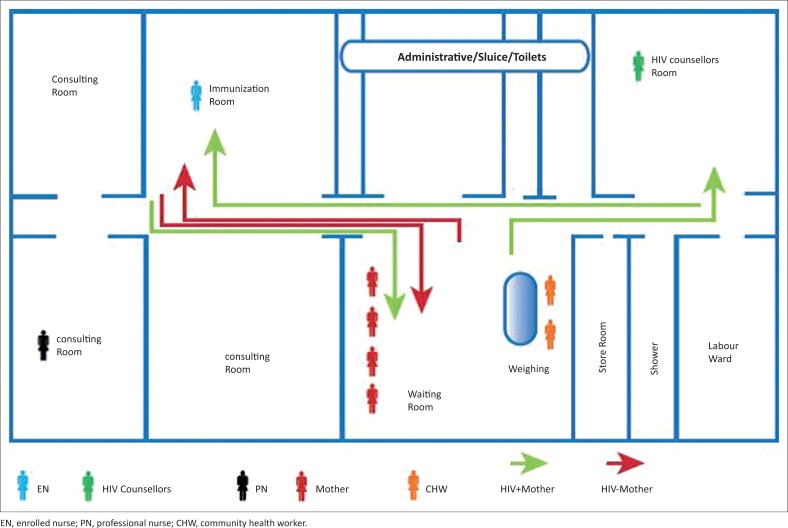
Layout of health facility and depicting consulting rooms and the movement of mothers.

Figure 1 illustrates the layout and organisation of services in one clinic. In this example community health workers directed mother–baby pairs around the clinic. They weighed babies in the waiting room, and asked mothers about their HIV status in the waiting room. Mothers who were HIV-positive were directed to the HIV counsellor before going to immunisation room where the enrolled nurse administered the immunisation. HIV-positive mothers often left the clinic without being seen by a professional nurse, thus bypassing essential post natal PMTCT services. If the mother was HIV-negative, the mother was directed to the immunisation room where the immunisation was administered after which the mother left the clinic without being seen by a professional nurse.

### Interviews with health workers

A total of 116 health workers were on duty at the time of data collection in the 12 clinics, and all were interviewed. Demographic details of health workers are shown in Table 3.

**TABLE 3:** The demographic characteristics of participating health workers.

Cadre of health worker: *N* = 116	Professional nurse *n* = 47	Enrolled nurse *n* = 32	HIV counsellors *n* = 29	Enrolled nurse assistant *n* = 8
Median age (IQR)	44 (35–53)	34.5(31–39)	35 (32–40)	31 (29–35)
Female (male)	42 (5)	7 (5)	21 (8)	8 (0)
Years worked at current facility Median (IQR)	6.6 (0–27)	2.8 (0–8)	4.8 (1–9)	1 (0–7)

IQR, Inter quintile range.

**FIGURE 2:**
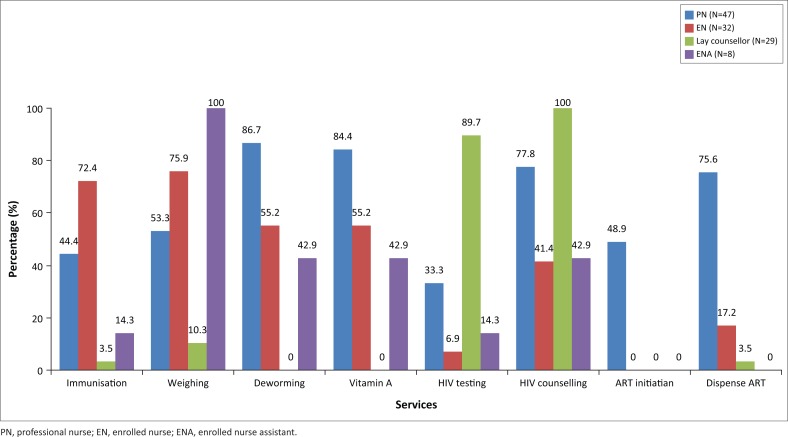
Services provided by different cadre of healthcare workers.

All health workers were asked about the services they provide to mothers and children. All health workers reported services they provided for mothers and children (Figure 2).

**FIGURE 3:**
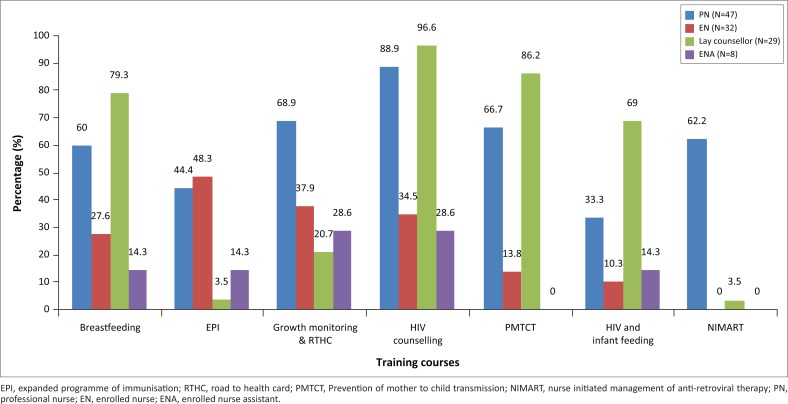
Training courses attend by different cadre of healthcare workers.

Enrolled nurses play a key role in providing child health services while HIV counsellors take the lead in the provision of HIV care. Health workers reported on maternal and child health training courses they had attended relevant to the services they provide, which were available through the Department of Health (Figure 3).

### Exit interviews with mothers

**FIGURE 4:**
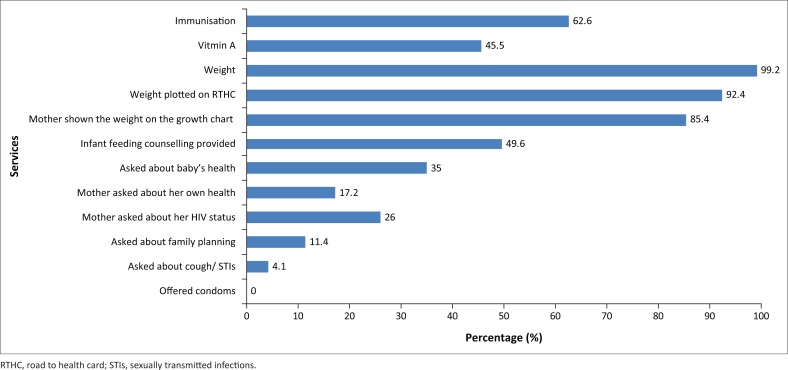
Services received by mothers on well-child visit.

A total of 211 mothers were interviewed upon exiting the clinic. The median age of mothers was 24.5 years (IQR 21–30 years), and median age of the baby was 5 months (IQR 2–9 months). Most mothers (92.4%) had at least some high school education, but few mothers were employed (14.2%). Although almost every baby was weighed and plotted on the Road to health card (RTHC), fewer than half of mothers received advice or were asked about their baby’s health (Figure 4). When compared to HIV-negative mothers, HIV-positive mothers were more likely to be asked about their baby’s health (44.9% vs. 35.1%), were more likely to be able to speak to a health worker privately (30.8% vs. 19.1%), and were more likely to be given advice on infant feeding (52.6% vs. 26.7%). A variety of services were provided to mothers and babies on this well-child visit as reported by the mothers (Figure 4).

## Discussion

This study highlights many missed opportunities for health workers to provide comprehensive integrated MCWH care to mothers and babies attending well-child clinic visits. Although a broad range of essential services for mothers and babies and most equipment needed to provide these services was available at the clinic, mothers and babies frequently left the clinic without receiving comprehensive care.

Fragmentation of services occurred as a result of different services being provided by different health workers in different rooms comparable to the findings described by Uebel, Guise, Georgeu, Colvin and Lewin.^5^ Most mothers had to either return on another day or go to another consulting room to access services. However, mothers were often reluctant to wait for a second consultation therefore their other option was to return on another day. This was also not favoured since it was expensive, time-consuming and inefficient for both mothers and health workers.^17^ Similar findings are described elsewhere, where health workers providing essential MCWH care were often situated far from the immunisation room and mothers either had to be referred to these health workers, or had to request these services themselves.^18^ Additionally, most equipment required by nurses was not available in the immunisation room where mothers and babies received well-child services, and mapping showed that the professional nurse was often bypassed in the designated flow that mothers followed through the clinic. This also led to fragmentation of service provision, with mothers systematically missing out on services within the scope of practise of professional nurses, including prescribing of medication. To ensure all mothers and babies receive a full package of care when attending for well-child services, equipment should be centralised and/or available in all rooms where services are provided.

Fragmentation of services has been criticised for increasing cost, providing poor quality of care and fostering poor health outcomes.^19,20,21^ Even in developed countries, patients are more dissatisfied with healthcare within systems where fragmentation is greater.^22^ To address fragmentation, there needs to be greater service integration of essential MCWH care. Ideally this should be a comprehensive package of care that is seamless, smooth and easily navigable by mothers and their babies. Patients want an organised coordinated service, where the number of visits to the facility, and the number of stages in a visit, are minimised to reduce waiting times.^23^ Although integration can mean different things to different people, and may require different models in different settings, the basic premise of easily accessible, continuous and comprehensive care remains a common thread. However, integration of services has been difficult to achieve, scale up and sustain.^23,24^

In regards to HIV care in South Africa paediatric HIV disease is responsible for more than half of child deaths, and current guidelines require early infant HIV diagnosis, with initiation of ART at the time of HIV diagnosis.^25^ However, loss to follow up for HIV care in the postnatal period remains a challenge leading to high mortality among HIV-infected infants^4,5,26^ despite high immunisation coverage over the same time period. Thus, by integrating postnatal HIV care with well-child services, health policymakers and practitioners can help actively reduce the burden of paediatric HIV disease.

The roles of health workers in providing a comprehensive package of care for mothers and babies need to be clearly defined and optimised within the designated scope of practise for each cadre of health worker. This study showed that enrolled nurses played a central role in the provision of well-child services, but many training courses are attended by professional nurses (PNs) and HIV counsellors with relatively little training being aimed at enrolled nurses. Enrolled nurses should receive up-to-date, appropriate training in all services within their scope of practice, as well as being knowledgeable about indications for services not within their scope of practise, so that gaps and opportunities for service provision are not missed. Currently, HIV counsellors only see HIV-positive mothers; thus, HIV-negative mothers are missed and do not receive appropriate counselling or retesting for HIV. This also results in opportunities for mothers to miss out on HIV care or for inadvertent disclosure of HIV status as described in previous studies.^5^ Therefore, HIV counsellors should see all mother–baby pairs.

Furthermore, ethical and confidentiality issues were noted in most clinics. Mothers had limited opportunity to privately consult with health workers during the clinic visit. Mothers and babies should have been seen privately and weighing should be done privately, rather than in a public space with other mothers and babies present in accordance with recommended core standards,^12^ to allow mothers the opportunity to discuss personal issues. In addition, it is important for healthcare workers to thoroughly examine the child while they are undressed.

In a South African setting, where several health workers collaborate to provide services, integrating the full range of mother, child, and PMTCT services is complex and difficult. Skilled health workers are scarce so human resources have to be used with maximum efficiency. Integration involves developing models suitable for this context, which may even vary from clinic to clinic according to space and staffing, to provide, as far as possible, a seamless, user-friendly service. Health workers will have to change long-established practices and overcome challenges to provide a full package of care for mothers and their babies.

### Strengths and limitations

This study was conducted in a small number of facilities in a single district on a particular day and may not represent service provision in other districts. However, the findings were similar across all clinics visited and supported findings of other studies.

The use of self-reported data relies on subjective recall of events and this may not be a true representation of what has happened. In addition, interviews were undertaken in a health facility, so that participants may have been reluctant to be critical about service provision in the facility.

## Conclusion

Strengthening MCWH services in PHC clinics is essential to reduce maternal and child morbidity and mortality in South Africa. Despite availability of most essential services in the clinics, extensive gaps remain in the provision of comprehensive care for mothers and babies in the postnatal period. Mothers were forced to visit clinics multiple times to receive all services because of ineffective organisational flow of patients through the clinics and the misallocation of supplies and equipment. Providing integrated MCWH and HIV service during well-child visits is an opportunity to improve outcomes for mothers and babies by ensuring they receive a comprehensive package of follow up care in the postnatal period, and can reduce multiple clinic visits and make services more accessible for all mothers and babies.

### Recommendations

Based on the findings of this study, we make the following recommendations:

- Change the flow of patients through the clinic to provide mother–baby pairs a full package of comprehensive MCWH services, by the least number of health workers, without moving from room to room.
- All mother–baby pairs consult with a professional nurse at every well-child visit.
- Essential equipment and supplies should be available in the consulting room where mother–baby pairs are seen.
- HIV care is available to all mother–baby pairs, regardless of the HIV status.
- Roles and responsibilities for health workers providing a full package of comprehensive care are clearly defined and followed.
- Privacy and a place to talk confidentially with mother–baby pairs are provided at all times.
